# Head Acceleration Event Exposure During Elite Men’s and Women’s Rugby Union Training

**DOI:** 10.1007/s40279-025-02287-2

**Published:** 2025-08-02

**Authors:** Samuel Hudson, James Tooby, Gregory Roe, Thomas Sawczuk, Dario Cazzola, Matt Cross, Ben Jones, Simon Kemp, Sarah Whitehead, Keith Stokes

**Affiliations:** 1 Centre for Health and Injury and Illness Prevention in Sport, University of Bath, Bath, UK; 2 UK Collaborating Centre on Injury and Illness Prevention in Sport (UKCCIIS), University of Bath, Bath, UK; 3 Carnegie Applied Rugby Research (CARR) Centre, Carnegie School of Sport, Leeds Beckett University, Leeds, UK; 4 Obesity Institute, Leeds Beckett University, Leeds, UK; 5 Premiership Rugby, London, UK; 6 Division of Physiological Sciences, Department of Human Biology, Faculty of Health Sciences, University of Cape Town, Cape Town, South Africa; 7 School of Behavioural and Health Sciences, Faculty of Health Sciences, Australian Catholic University, Brisbane, QLD Australia; 8 Rugby Football League, Etihad Campus, Manchester, UK; 9 Rugby Football Union, Twickenham, UK; 10 Department of Epidemiology and Population Health, London School of Hygiene and Tropical Medicine, London, UK

## Abstract

**Objectives:**

The aim of this study was to describe the incidence and magnitude of head acceleration events (HAEs) during elite men’s and women’s rugby union training for different contact training levels and drill types.

**Method:**

Data were collected during the 2022–23 and 2023–24 seasons from 203 men and 125 women from 13 clubs using instrumented mouthguards (iMGs) during in-season training. One author reviewed the training videos to identify the contact level and drill type. HAE incidence was calculated per player minute.

**Results:**

For men’s forwards and backs, only 4.7% and 5.8% of HAEs were ≥ 25 g and ≥ 1.5 Krad/s^2^, and 3.4% and 4.4% for women’s forwards and backs, respectively. The incidence of ≥ 5 g and ≥ 0.4 Krad/s^2^ was highest during full-contact training for men’s forwards (0.20/min) and backs (0.16/min) and women’s forwards (0.10/min). HAE incidence was 2–3 times higher during repetition-based compared with game-based training drills for men’s forwards (0.25/min vs 0.09/min) and backs (0.22/min vs 0.09/min) and women’s forwards (0.09/min vs 0.04/min) and backs (0.08/min vs 0.03/min). HAE incidences were halved when repetition-based training drills used pads compared with no pads for men’s forwards (0.21/min vs 0.44/min) and backs (0.17/min vs 0.30/min), and women’s forwards (0.06/min vs 0.14/min) and backs (0.06/min vs 0.10/min).

**Conclusion:**

The average HAE incidence (~ 13–20% of weekly HAEs) and magnitude during an in-season training week is very low compared with matches. Opportunities to materially reduce HAE exposure in training are likely more limited than previously assumed. Future research on HAE load and injury, and understanding players’ specific weekly training exposure, may inform effective individual player management.

**Supplementary Information:**

The online version contains supplementary material available at 10.1007/s40279-025-02287-2.

## Key Points

**Table:** 

The count, incidence, and magnitude of HAEs during an elite men’s and women’s rugby union training week is low compared with matches.
League-wide interventions that aim to reduce HAE exposure in training are likely limited due to the necessity of contact for competition preparation, and an individualised approach.
Player welfare strategies should principally focus on reducing HAE exposure during matches.

## Introduction

Rugby union is a collision-based team sport involving bouts of high-intensity exercise [1] and contact events including tackles, ball carries, rucks, mauls, and scrums [2]. During contact events, players might experience head acceleration events (HAEs) [3, 4], which are defined as a sudden change in the speed of the head’s centre of mass induced by a short-duration collision from an external force directly to the head or indirectly via the body [5, 6]. Players may experience HAEs that do not result in the immediate presentation of clinical symptoms and/or a concussion diagnosis, but the medium- and longer-term implications are less understood. HAE accumulation has been associated with negative later-life brain health outcomes including cognitive deficits [7, 8], hazard of death [9], and neurodegenerative diseases [10]. Match HAE exposure has been quantified in elite rugby union matches [3, 11–15] and modelled in overall training compared with matches [15]. However, HAEs in training have not been investigated by contact-level, or for game-based and repetition-based training drills, despite the nature of these activities differing significantly from each other and players spending five times more of their rugby activity time in training than in matches [16].

Instrumented mouthguards (iMGs) have been recently validated [17] and thus it is now possible and recommended to count and measure the magnitude of HAEs [6, 18]. As a modifiable environment with a relatively higher degree of control compared with matches, identifying the frequency and magnitude of HAEs by specific training drills would support the development of data-informed player welfare strategies and optimal loading prescriptions that may reduce unnecessary HAE exposure. Therefore, this study aimed to describe the incidence and magnitude of HAEs during elite men’s and women’s rugby union training at different levels of contact training and in different types of drill.

## Method

### Study Design and Participants

A prospective observational cohort study was conducted with seven Gallagher Premiership men’s teams and six Premier 15 s/Premiership Women’s Rugby teams, who participate in the highest level of rugby union competition in England. Data were collected during 132 men’s and 69 women’s regular in-season training sessions during the 2022–23 (26 training sessions from four men’s teams, and 8 sessions from two women’s teams), and 2023–24 (106 training sessions from six men’s teams, and 61 training sessions from five women’s teams) seasons. Ethical approval was obtained from the Research Ethics Approval Committee for Health (EP 20/21 088). Across the 13 teams sampled, > 99% provided informed consent to take part in the study.

### Data Collection

Participants were fitted with a custom Prevent Biometrics iMG (Minneapolis, MN, USA) from 3D digital dental scans taken by an experienced dentist. The iMGs are equipped with an accelerometer and gyroscope, with measurement ranges of ± 200 g and ± 35 rad/s and a sampling rate of 3.2 kHz. Embedded in the iMG were infrared proximity sensors that detect coupling to the upper dentition to start monitoring head kinematics. The Prevent Biometrics iMG has been validated in both laboratory and on-field studies [19–21], with a concordance correlation coefficient of 0.98 for the laboratory accuracy of head kinematic measures, and positive predictive value of 0.94 and sensitivity of 0.75 for on-field video verification validation [19]. Only HAEs that exceeded both 5 g (at the head centre of gravity) and 400 rad/s^2^ were used per previous rugby iMG studies [3, 11–15]. This threshold helps to remove non-contact HAEs (e.g., running and jumping) which might be captured, with a positive predictive value of 0.99 (95% CI 0.97–1.00) [3].

A sensor acceleration event, which is the iMG datum that approximates HAEs at the head’s centre of gravity [22], was recorded by the iMG when one of the axes on the medial–lateral, anterior–posterior, and vertical planes exceeded 8 g, capturing 50 ms of head kinematics (10 ms pre-trigger; 40 ms post-trigger). The peak linear acceleration (PLA; g) and peak angular acceleration (PAA; rad/s^2^) of each event was calculated from resultant values. To account for tangential and acceleration effects [23], linear head kinematics were transformed to the 50th percentile male head centre of gravity from the iMG location. Prevent Biometrics’ in-house algorithm categorised signal noise for each HAE recorded as minimal (0; *n* = 5660), moderate (1; *n* = 374), or severe (2; *n* = 270). A 4-pole, zero-phase, low-pass Butterworth filter was employed, with class 0, 1, and 2 signals using 200, 100, and 50 Hz cut-off frequencies, respectively. This is consistent with previous research [3, 11–15].

Integrating video in wearable sensor studies is recommended to support data windowing when researching on-field head kinematics [5]. Consequently, a member of the research team attended each of the participating clubs for three consecutive training weeks to capture tight video angles from height at the central point of the pitch of all field-based training sessions. As clubs had access to iMGs for the entire season and collected data routinely, teams were asked to share their analyst recorded video footage of conventional, regular-season training sessions to supplement data collected by the researcher. All angles were timestamped with the real-world time that the video recording began in HHMMSS format. This allowed temporal data windowing [5] to take place. Training activities were determined by the lead author from the video of training and were allocated into one of the activity types in Table 1, adapted from Starling et al. [18]. Training activities fitted one of three defined levels of contact; *full contact* (live collisions between players in an uncontrolled environment), *controlled contact* (play in a controlled environment at reduced speed and/or intensity, either between players or through the incorporation of pads, shields, bags, and/or crash mats), and *non-contact* (unopposed play with no intensity or force in contact or collisions between players). Game-based training drills, characterised by 15v15 match play simulation, were observed at all three levels of contact. Repetition-based training drills (live breakdown, live tackle drill, wrestling drill; all without the use of pads, and breakdown bags opposed, breakdown bags unopposed, tackle bag drill; all with pads) were all categorised as controlled-contact level, apart from passing drills (non-contact). The real-world start and end times of drills, defined as the duration between the start of the first set or repetition and the end of the last, were recorded as used as the training exposure duration. Simultaneously occurring activities were assessed separately to recognise potential differences in level of contact and drill type. For example, a three-station contact skills rotation was separated into nine training drills. Only training drills that were captured on video for their entirety during sampled training sessions were included in the analysis. Data from training sessions where there was no video shared, or where the video timestamp did not align with HAEs in video, were excluded from the study.

**Table 1:** Definitions and categorisation of contact level and the training drills sampled

Contact level	Drill level	Drill type	Drill type definition
Full-contact (live collisions between players in an uncontrolled environment)	Game-based	Team full contact	15 vs 15 at match intensity
	Units	Live lineout	Mauling lineout set pieces at match intensity
		Live scrum	Scrummaging at match intensity
		Live backs units	Backs full contact matchplay drill
Controlled-contact (collisions between players in a controlled environment at reduced speed an/or intensity)	Game-based	Team shoulder on	15 vs 15 bone-on-bone at a reduced intensity
		Team vs pads	15 vs 15 with defenders holding pads
	Repetition-based (without bags)	Live breakdown	Rucking in a controlled space
		Live tackle drill	Bone-on-bone tackling in a controlled space
		Wrestling	Scrag drill
	Repetition-based (with bags)	Breakdown bag (opposed)	Rucking against a pad, with weight behind
		Breakdown bag (unopposed)	Rucking against a pad, without weight behind
		Tackle bag drill	Tackling against a bag in a controlled space
	Units	Lineout vs pads	Mauling lineout set pieces against pads
		Sled scrum	Scrummaging against a sled
Non-contact (unopposed play with no intensity or force in contact or collisions between players)	Game-based	Team unopposed organisation	15 vs 15 with no intensity in player collisions
	Units	Unopposed lineout	Non-mauling lineout set pieces
		Unopposed backs units	Backs unopposed strike plays
	Repetition-based	Passing	Drills focusing on handling skills

### Time on Teeth

**Fig. 1:**
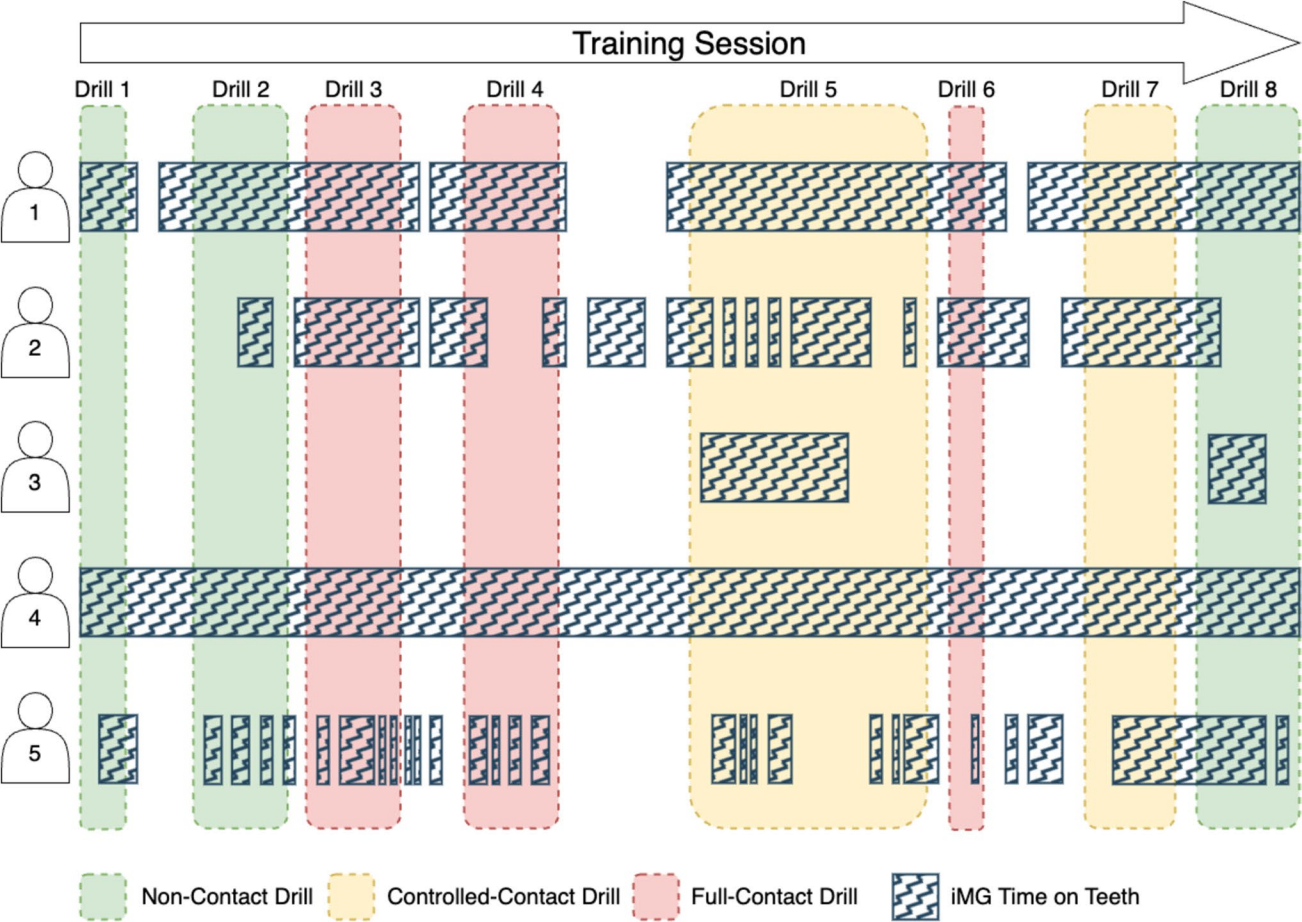
An example visualisation of iMG time on teeth throughout a training session. Vertical shaded areas indicate the type of training drill and when it occurs during the training session. Horizontal zigzagged bars indicate when the iMG is being worn by players, and the gaps between them when it is not, during the training session. A player is assumed to have worn their iMG for all their involvements in a drill, and therefore all potential HAEs are captured if they have at least one time-on-teeth log in a drill. They are included in the training exposure calculation. *HAEs* head acceleration events, *iMG* instrumented mouthguard

Players were asked to wear their iMG from the start of training, and refrain from removing their iMG until the end of training drills, to ensure all potential HAEs were captured for each training activity (Fig. 1). Therefore, iMG adherence when actively participating in training activities was visually inspected in a sample of players and training videos used in this study to determine whether all contact events were monitored for HAEs. A sample of 47 players (24 men [16 forwards and 8 backs] and 23 women [18 forwards and 5 backs]) were followed through 51 men’s and 45 women’s player-training sessions across four men’s and three women’s clubs, contributing to 368 men’s and 237 women’s player drill exposures. Some players were observed to not wear their iMG at all for some training drills during the training sessions sampled. However, during the training drills in which players did wear their iMG, only five men’s player-drill exposures (< 1% of player-drill exposures) were observed where a player did not wear their iMG for all contact events they engaged in. Players otherwise only removed their iMG during training drills when they were not actively participating in the drill (e.g., between repetitions). A similar visual inspection of iMG adherence behaviours was also tested in another study of HAE exposure during elite men’s and women’s rugby union training using iMGs on a smaller sample of the same population [15]. Roe et al. [15] reported that 100% of player-drill observations inspected saw iMGs worn for all contact events, and thus generalised iMG adherence behaviours to their whole study sample. Therefore, they assumed that a player had worn their iMG for their whole involvement in the training drill if they had at least one time-on-teeth log in its duration. Due to the overwhelming proportion of player exposures observed to have worn their iMG for their involvement during a training drill in this study and previously in the literature [15], the same assumption was made, and players with at least one time-on-teeth log during a training drill were included in the training exposure calculation. Training exposure was defined as the sum of drill durations. This was assessed on a player-by-player basis as their inclusion in calculations depended on whether they wore their iMG in each training drill. A custom-written MATLAB script was used to visualise time-on-teeth during training drills to identify the number of player exposures for each training activity.

### Statistical Analysis

The incidence of HAEs was calculated per player minute for each player during each contact level and each drill type that they participated in by dividing the total HAE count by total minutes of training exposure. The overall HAE incidence for each contact level and drill type was calculated by averaging the rates for all players. This was to reduce the bias of individual player variability in HAE count and training exposure and is consistent with HAE rugby match exposure research [3] and the consensus statement for epidemiological data reporting [24]. The incidence of HAEs was calculated for those at the minimum threshold (≥ 5 g and 0.4 Krad/s^2^) and for ≥ 25 g and ≥ 1.5 Krad/s^2^, to allow comparison with the literature for higher magnitude HAEs. The HAE and training exposure data were organised in Microsoft Excel (version 16.89.1) and read into R (version 4.3.1) for analysis in R Studio (version 2023.12.1 + 402). Confidence intervals at 95% confidence were estimated using bootstrapping, and resampling 1000 times as player exposures for different drills varied from 0.4 to 641.1 min.

## Results

**Fig. 2:**
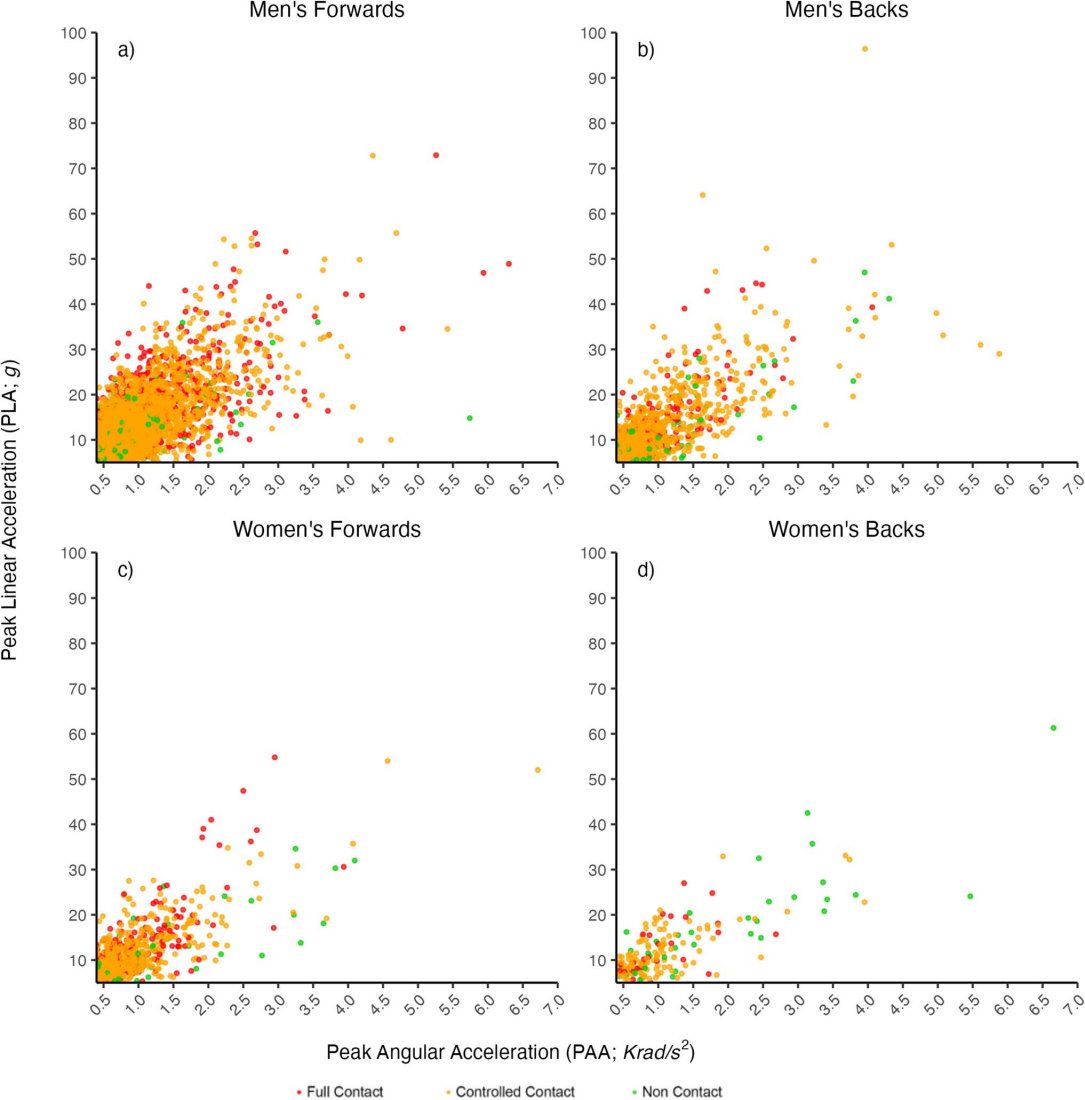
The magnitude of head acceleration events (HAEs) in each level of contact training for men’s and women’s forwards and backs

Incidence values were derived from 203 male and 125 female players, including 8160 and 2532 player–drill exposures (941 and 365 player hours of training exposure) (Table S1 in the electronic supplementary material [ESM]) across 132 and 69 training sessions, respectively. Only 62% of male and 53% of female players who provided informed consent wore their iMGs during the sampled training sessions. Time-on-teeth logs for iMGs indicated that removal during training drills was common. There were 4289 men’s forwards HAEs recorded ≥ 5 g and ≥ 0.4 Krad/s^2^ (Fig. 2). Of these, 200 (4.7%) were ≥ 25 g and ≥ 1.5 Krad/s^2^, and 1 (< 0.1%) was ≥ 50 g and ≥ 3.0 Krad/s^2^. There were 1131 men’s backs HAEs recorded ≥ 5 g and ≥ 0.4 Krad/s^2^. Of these, 66 (5.8%) were ≥ 25 g and ≥ 1.5 Krad/s^2^, and 2 (< 0.1%) were ≥ 50 g and ≥ 3.0 Krad/s^2^. There were 701 women’s forwards HAEs recorded ≥ 5 g and ≥ 0.4 Krad/s^2^. Of these, 24 (3.4%) were ≥ 25 g and ≥ 1.5 Krad/s^2^, and 1 (0.1%) was ≥ 50 g and ≥ 3.0 Krad/s^2^. There were 183 women’s backs HAEs recorded ≥ 5 g and ≥ 0.4 Krad/s^2^. Of these, 8 (4.4%) were ≥ 25 g and ≥ 1.5 Krad/s^2^, and 1 (0.5%) was ≥ 50 g and ≥ 3.0 Krad/s^2^.

### Contact Level

The mean incidence of HAEs ≥ 5 g and ≥ 0.4 Krad/s^2^ per player minute was twice as high in men’s full-contact training compared with women’s. Men’s forwards full-contact training had a higher mean incidence of HAEs per player minute (0.20/min [95% CI 0.16–0.26]) than backs (0.16/min) [0.09–0.41]). The highest incidence of HAEs per player minute in women’s forwards training was in full-contact training (0.10/min [0.08–0.13]). The incidence of HAEs per player minute in women’s backs full-contact training was 0.05/min [0.03–0.10], which was the same as controlled-contact training (0.05/min [0.04–0.07]). Women’s forwards had a similar HAE incidence per player minute of 0.05/min [0.04–0.06] during controlled-contact training, whilst men’s forwards and backs HAE incidence per player minute in controlled-contact training was twice as high (0.09/min [0.08–0.10]; 0.11/min [0.08–0.21], respectively). Compared with other contact levels, the incidence of HAEs per player minute was lowest during non-contact training for men’s forwards (0.03/min [0.02–0.05]) and backs (0.02/min [0.01–0.03]), and women’s forwards (0.01/min [0.01–0.01]) and backs (0.04/min [0.02–0.07]. The incidence of HAEs ≥ 25 g was 0.02/min in men’s forwards [0.01–0.03] and women’s backs [0.00–0.05] full-contact training. The incidence of HAEs ≥ 25 g was 0.01/min [0.00–0.01] in men’s backs and women’s forwards full-contact training. The incidence of HAEs ≥ 25 g was 0.01/min in men’s forwards [0.00–0.01] and men’s backs [0.00–0.02] in controlled-contact training. The incidence of HAEs ≥ 1.5 Krad/s^2^ was 0.02/min in men’s forwards [0.02–0.03] and 0.01/min [0.01–0.02] in men’s backs full-contact training, and 0.01/min [0.01–0.02] in men’s forwards and 0.02/min [0.01–0.04] in men’s backs controlled-contact training. The incidence of HAEs ≥ 1.5 Krad/s^2^ was 0.01/min for women’s forwards [0.01–0.02] and backs [0.00–0.01] in full-contact training, and in controlled-contact training [0.00–0.01, and 0.00–0.02, respectively].

**Fig. 3:**
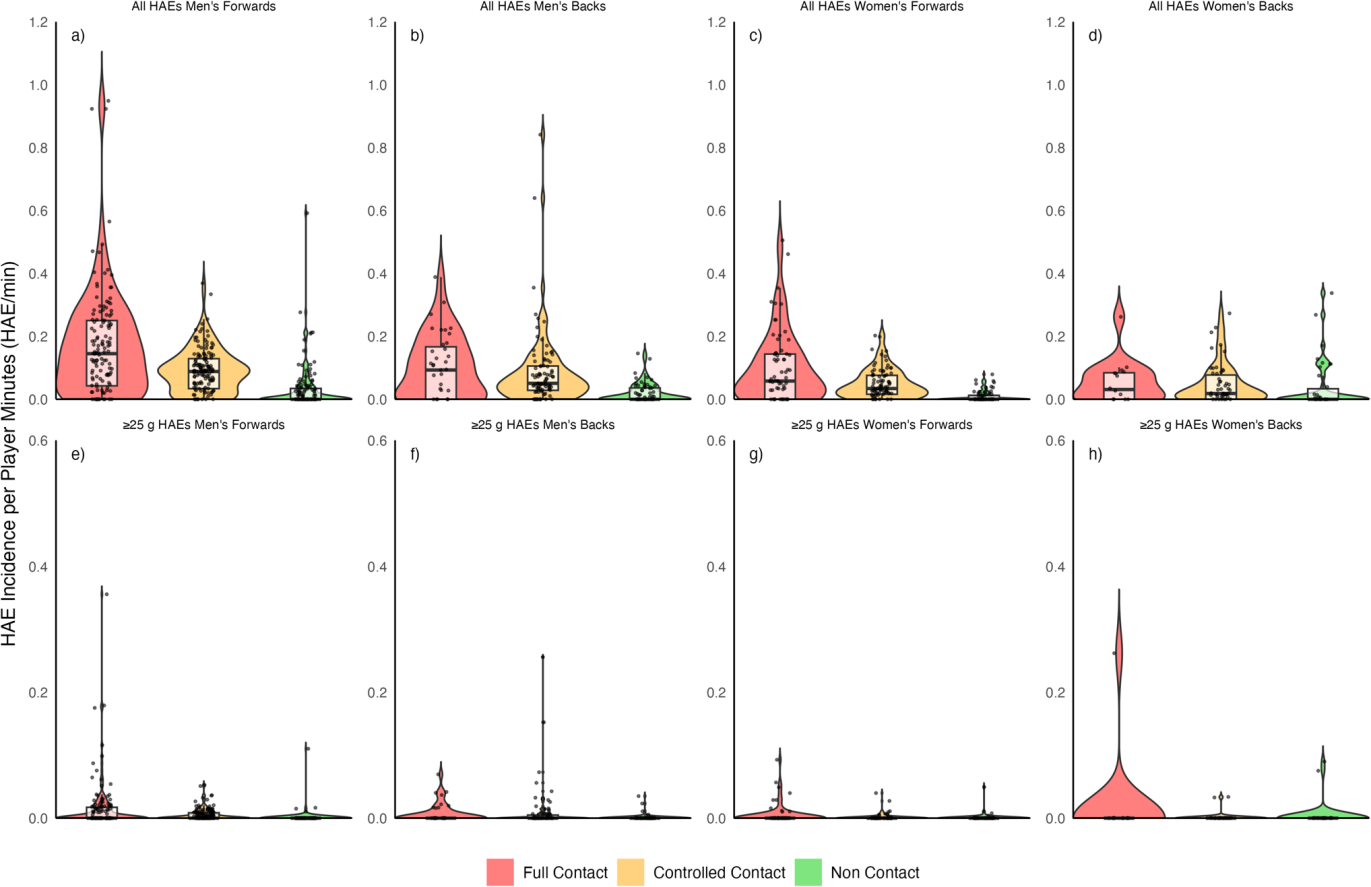
Violin plot of head acceleration event (HAE) incidence per player minute by level of contact training. The *Y* axes scale for ≥ 25 g HAEs (**e**–**h**) is half of that for the all-HAEs (**a**–**d**) plots

For men’s forwards and backs, and women’s forwards, the largest interquartile range of HAE incidence per player minute values was observed during full-contact training (Fig. 3). However, the median HAE incidence per player minute was similar in full-contact and controlled-contact training. In women’s backs, the interquartile range and median of HAE incidence per player minute in full-contact and controlled-contact training were the same.

### Drill Level

**Fig. 4:**
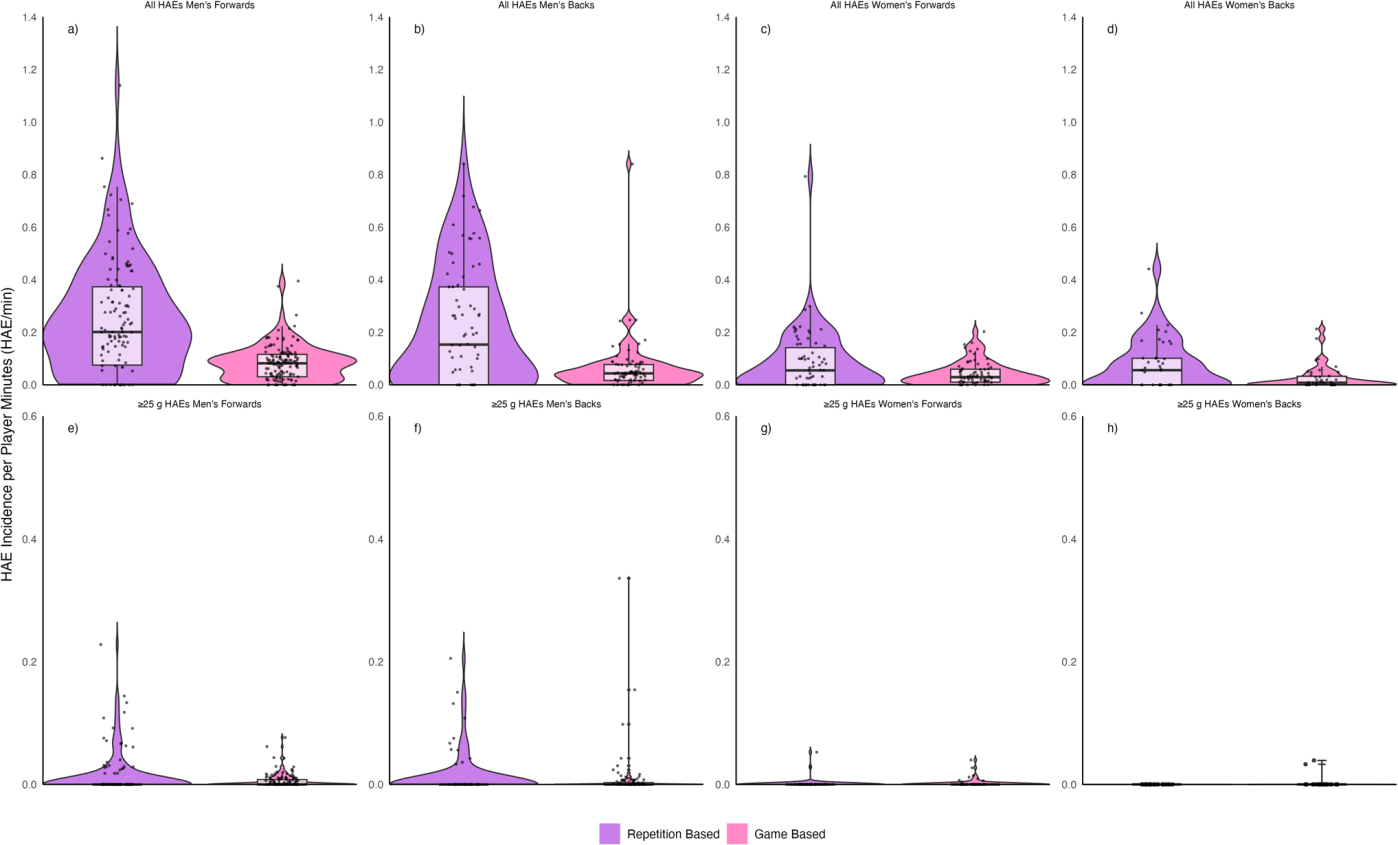
Violin plot of head acceleration event (HAE) incidence per player minute during repetition-based and game-based training drills. The *Y* axes scale for ≥ 25 g HAEs (**e**–**h**) is different to the all-HAEs (**a**–**d**) plots

The incidence of HAEs ≥ 5 g and ≥ 0.4 Krad/s^2^ per player minute was two to three times higher during repetition-based training drills compared with game-based training drills for men’s forwards (0.25/min [0.21–0.29] vs 0.08/min [0.07–0.09]), men’s backs (0.22/min [0.17–0.27] vs 0.09/min [0.05–0.19]), women’s forwards (0.09/min [0.06–0.12] vs 0.04/min [0.03–0.06]), and women’s backs (0.08/min [0.05–0.12] vs 0.03/min [0.02–0.05]). The incidence of HAEs ≥ 25 g was 0.01/min in repetition-based and game-based training drills for both men’s forwards ([0.01–0.02] and [0.00–0.01], respectively) and men’s backs ([0.01–0.03] and [0.00–0.03], respectively). The incidence of HAEs ≥ 1.5 Krad/s^2^ during repetition-based training drills was 0.03/min [0.02–0.05] for men’s forwards and backs, and 0.01/min for women’s forwards [0.00–0.01] and backs [0.00–0.02]. The incidence of HAEs ≥ 1.5 Krad/s^2^ during game-based training drills was 0.01/min [0.0–0.02] for men’s forwards and 0.02/min [0.01–0.03] for men’s backs. The incidence of HAEs ≥ 1.5 Krad/s^2^ during game-based training drills was 0.01/min for women’s forwards [0.00–0.01] and backs [0.00–0.02]. The interquartile range of HAE incidence per player minute was wider during repetition-based training drills than game-based training drills (Fig. 4).

**Fig. 5:**
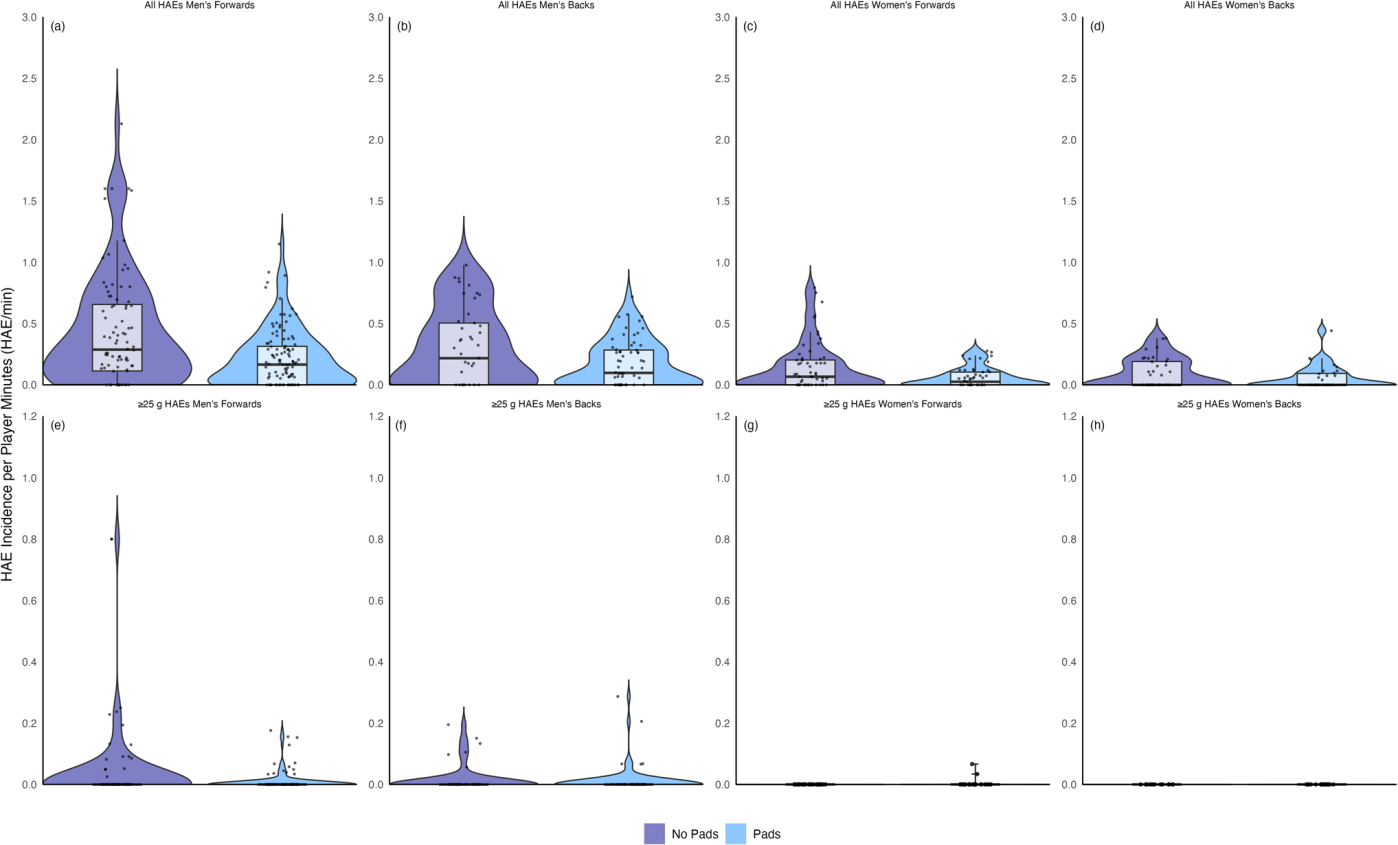
Violin plot of head acceleration event (HAE) incidence per player minute during repetition-based training drills without and with the use of pads. The *Y* axes scale for ≥ 25 g HAEs (**e**–**h**) is different to the all-HAEs (**a**–**d**) plots

When comparing repetition-based training drills, those that incorporated pads had half the HAE incidence ≥ 5 g and ≥ 0.4 Krad/s^2^ per player minute than those without pads. For men’s forwards, repetition-based training drills without pads had a HAE incidence per player minute of 0.44/min [0.35–0.54], twice as high as the 0.21/min [0.17–0.26] for repetition-based drills that included pads. Men’s backs had a HAE incidence per player minute of 0.30/min [0.22–0.40] in repetition-based training drills without pads, compared with 0.17/min [0.13–0.22] including pads. The incidence of HAE per player minute in women’s forwards repetition-based training drills which did not include pads was 0.14/min [0.10–0.20], compared with 0.06/min [0.04–0.09] when pads were included. For women’s backs, the HAE incidence per player minute in repetition-based training drills when pads were not used was 0.10/min [0.06–0.14], compared with 0.06/min [0.03–0.10] when pads were incorporated. The incidence of HAEs ≥ 25 g during repetition-based training drills without pads was 0.04/min [0.02–0.07] for men’s forwards and 0.02/min [0.01–0.03] for men’s backs. The incidence of HAEs ≥ 25 g during repetition-based training drills with pads was 0.01/min [0.00–0.03] for men’s forwards, and 0.01/min [0.00–0.02] for men’s backs. The incidence of HAEs ≥ 1.5 Krad/s^2^ during repetition-based training drills without pads was 0.09/min [0.06–0.15] for men’s forwards, and 0.04/min [0.02–0.07] for men’s backs. The incidence of HAEs ≥ 1.5 Krad/s^2^ during repetition-based training drills without pads was 0.01/min for women’s forwards [0.01 to 0.03] and backs [0.00 to 0.03]. The incidence of HAEs ≥ 1.5 Krad/s^2^ during repetition-based training drills with pads was 0.02/min for men’s forwards [0.01 to 0.03] and 0.03/min [0.02 to 0.06] for men’s backs. The interquartile range of HAE incidence per player minute was wider during repetition-based training drills that did not incorporate pads compared with those that did (Fig. 5).

## Discussion

This study described the incidence of HAEs in elite men’s and women’s rugby union in-season training by level of contact training and drill type, with overall low HAE counts, incidences, and magnitudes observed. For men’s forwards and backs and women’s forwards, the incidence of HAEs per player minute was highest in full-contact training, followed by controlled-contact and non-contact training. Repetition-based training drills observed an HAE incidence per player minute two to three times higher than during controlled-contact game-based training drills. Although already low, the incidence of HAEs per player minute during repetition-based drills was halved on average when pads were introduced. The average incidence of HAEs per player minute ≥ 25 g was ≤ 0.04 for men’s and women’s forwards and backs across all training conditions.

On average, a men’s forward and back would have to train at full-contact intensity for 5 and 6 min, respectively, to record one HAE at the lowest magnitude threshold, which is lower than reported in matches (44 and 26 per match for forwards and backs) [3]. Based on the findings of this study, if men’s players were to train according to World Rugby’s contact training guidelines of a maximum of 15 min of full-contact training and 40 min of controlled-contact training [26], men’s forwards and backs would experience on average three and two HAEs per week during full-contact training, and both would experience four HAEs in controlled-contact training per week. Women’s forwards and backs would experience on average one HAE in full-contact training, and two HAEs in controlled-contact training per week. World Rugby’s contact training guidelines also suggest a maximum of 30 min of live units training per week [25]. The incidence of HAEs per player minute in live scrum and live lineout training differed significantly (Supplementary Table 1 in the ESM) and therefore, depending on the proportion of time teams dedicate to each set piece type, the weekly HAE exposure in training can vary. However, the absolute count of HAEs in the training week relative to matches would still be low.

The study showed that overall HAEs in training are low; however, training is a modifiable environment for which coaches have a higher degree of control [26]. Whilst HAE mitigation strategies should materially also focus on matches, where the incidence and magnitude are higher, it might be easier to implement player welfare strategies in training. Given players are required to undertake contact training to minimise injury risk in matches and develop proficiency in contact events for performance purposes, a degree of HAE exposure in training is likely unavoidable. Consequently, the scope for HAE mitigation strategies in training might be more limited than previously assumed, necessitating a nuanced approach to balance preparation for competition and achieving the minimal effective contact dose and associated HAE exposure. This is likely to be highly individualised and applying mitigation strategies to the entire cohort such as limiting training time might be overly restrictive from a performance perspective [27] and might also inefficiently reduce HAE exposure for all players from a welfare perspective, dependant on where such a limit is set. Future research might aim to understand the relationship between HAE load and concussion injury diagnosis and to identify how HAEs occur in training.

**Fig. 6:**
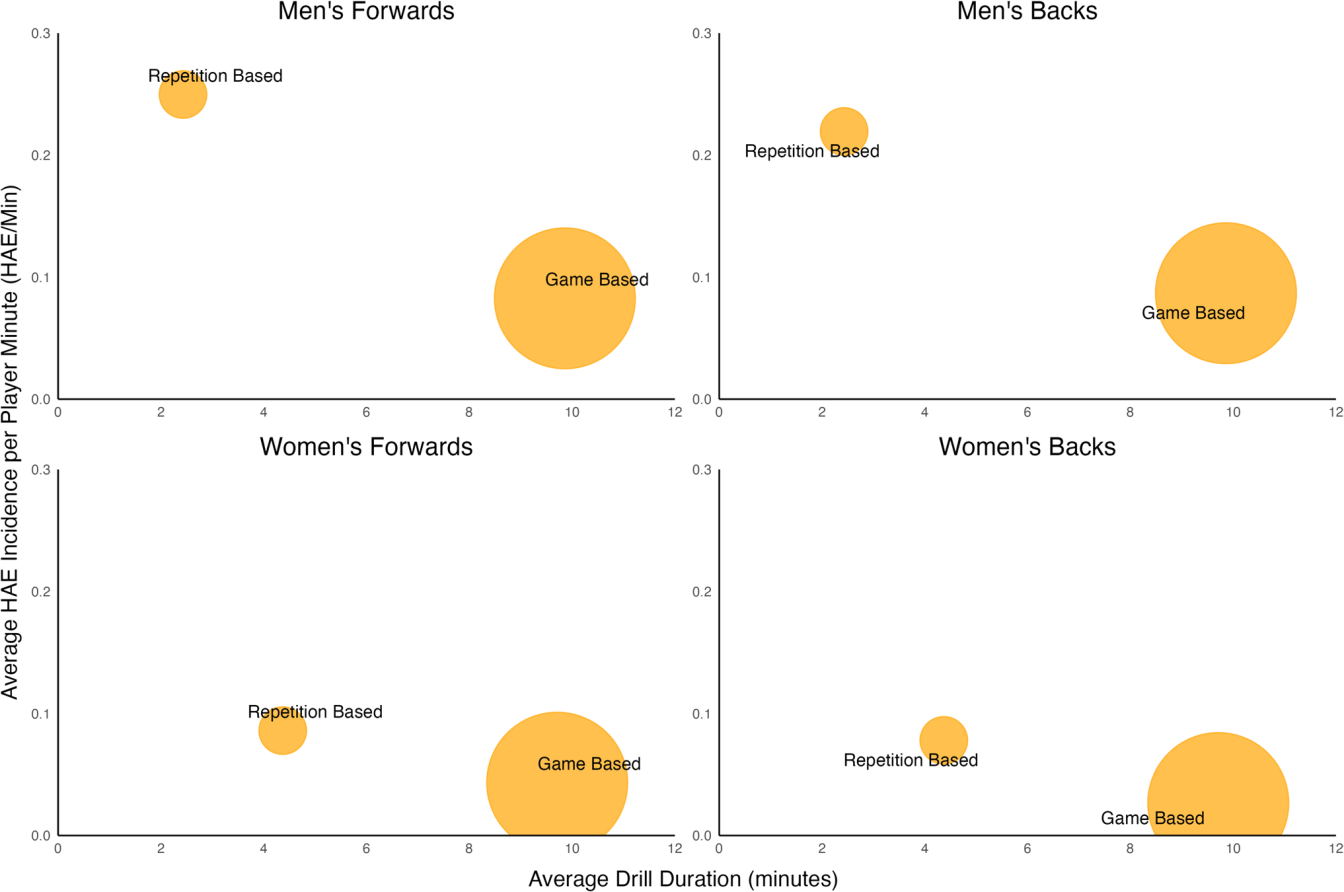
The incidence of head acceleration events (HAEs) per player minute for men’s and women’s forwards and backs in repetition-based and game-based training drills relative to their average duration in this study. The size of the bubbles represents the product of incidence and average drill duration

In this study, the incidence of HAEs during repetition-based training drills was two to three times higher than in controlled-contact game-based training drills. The higher incidence in repetition-based training drills is likely due to an increased contact event density for individuals. During repetition-based training drills, players repeatedly engage in an equal number of contact events in small groups over a short duration of time. In contrast, during game-based training drills, the frequency of contact event execution is sparse, due to having up to 30 players participating at any given time and depending on team strategy and position-specific demands. As such, there is a higher exposure to events that might incite HAEs during repetition-based training drills. However, most player training exposures were during game-based training activities, particularly at the controlled-contact level. In this sample, 38% of men’s and 45% of women’s player-training exposures were during game-based training drills at a controlled-contact level. When repetition-based training drills were performed, their duration was 5 times and 2.5 times shorter on average than game-based training drills for men and women, respectively (Fig. 6). Therefore, the low incidence of HAEs in training could be attributed to repetition-based training drills accounting for a minority proportion of players’ total training time. As such, current coaching practices might be broadly appropriate for balancing the mitigation of HAE exposure in training with team and player preparation.

During repetition-based training drills that incorporated pads, the incidence of HAEs was halved compared with when no pads were used. The contact skills performed between repetition-based training drills with and without pads included both the tackle and ruck, and the nature of the drills were comparable. Therefore, the use of pads might have a protective effect against HAE exposure. Whilst HAE incidence is already low, coaches might wish to introduce pads to repetition-based training activities to further minimise the exposure to HAEs during these activities.

The iMG data in this study contradicted players’ perceived risk of HAEs associated with training drills, particularly those involving contact. Starling et al. [18] reported that players described game-based training drills as possessing the highest risk of HAE exposure in training activities, despite the HAE incidence in this study being half that of repetition-based training drills. This difference might be due to players spending a higher proportion of their training time in game-based training drills (Fig. 6), and potentially lower player arousal to offset the pain of collisions in training simulations of match play, compared with competition, which has been reported in elite rugby league [28]. Future research should investigate the specific drills that contribute to a player’s typical weekly training exposure to enhance the accuracy of HAE exposure projections.

## Limitations

This is the first study to quantify the HAE exposure during different levels of contact and drill types in elite rugby union training. However, it is not without its limitations, such as the low player training exposure. The training sessions and drills sampled outside of the in-person three-week data collection blocks at each club were limited to those for which training footage was shared. Most training videos were of the club’s two main weekly training sessions and were largely composed of game-based training drills. Thus, smaller contact skills and units sessions that might have been dispersed throughout the week made up a lesser proportion of the sample. However, the training sessions that were sampled were representative of a typical ‘main’ session that clubs will have twice each week. A single coder of the contact levels and drill types in videos of training might introduce potential personal bias. As such, inter-rater reliability could not be assessed. Furthermore, lower-than-desirable iMG adherence limited the number of players in training for whom head kinematics were continuously monitored. Despite being encouraged to wear their iMG during sampled training sessions, ~ one-third of men’s players and ~ half of women’s players who provided informed consent did not wear their iMG during sampled training sessions. Unlike other wearable sensors routinely used in professional rugby such as GPS, which sits externally on the thoracic region of the spine, the iMG is more invasive and might be treated like a regular mouthguard for which low usage in training compared with matches has been reported [29]. A barrier to compliance has previously been reported due to the thickness of the iMG [30], despite performing well for comfort in a validation study [17]. It is unknown whether the proportion of players who did not wear their iMG during sampled training sessions chose not to, or whether they did not take part in those sessions. Removing player exposures and iMG data for those with incomplete time-on-teeth during training drills would have removed most data. Although player iMG time-on-teeth inspection in video showed that almost all player–drill observations saw all contact events were monitored for HAEs, it should be acknowledged that the assumption for training exposure calculation inclusion is based on a small sub-sample of the study’s participants and might not be generalisable to the entire cohort. Therefore, it is possible that an unknown degree of type II error might exist in the HAE dataset.

Furthermore, the limitations of the iMG devices should be recognised. The HAE exposure reported in this study is likely underestimated [24] due to the potential linear acceleration bias [31]. In their study, Wang et al. [31] demonstrated how 75% of 10 g HAEs and 14% of 20 g HAEs at the head’s centre of gravity were not captured by an iMG with a 10 g trigger threshold at the sensor location. Despite validation of the in-house Prevent Biometrics head kinematic filters as part of studies assessing the iMG system as a whole [19–21], these components have not been investigated in isolation from one another, nor have the iMG proximity sensors that detect coupling to the upper dentition. Finally, only the peak head kinematic in the 40 ms after the 8 g trigger threshold exceeded was used. Thus, pulse duration was not examined, which likely affects brain strain and injury risk [32].

## Conclusion

This study is the first to quantify HAEs during in-season training, consisting of different levels of contact training and drill types in elite men’s and women’s rugby union. The overall exposure and magnitudes of HAEs in this study were low, with high magnitude HAEs ≥ 25 g and ≥ 1.5 Krad/s^2^ rare compared with matches. The incidence of HAEs was twice as high in men’s training than women’s. Full-contact training HAE incidence was twice as high than controlled-contact training, which was higher than non-contact for men’s forwards and backs and women’s forwards. The incidence of HAEs was two to three times higher in repetition-based training drills compared with game-based training drills, and the incorporation of pads into repetition-based training drills halved the incidence of HAEs compared with drills without their inclusion. However, full-contact training (14% of men’s, 12% of women’s) and repetition-based training drills (10% of men’s, 23% of women’s) made up a minority proportion of players’ total training time. When multiplying HAE incidence per player minute to the equivalent minutes spent in different levels of contact training according to World Rugby’s contact training guidelines, this study suggests that the average total HAE count in a training week is very low. Therefore, the opportunity for reducing HAE exposure is likely to be limited in training, and mitigation strategies should principally focus on matches. Nevertheless, coaches and practitioners can further reduce HAE exposure by delivering game-based training drills and using pads when coaching repetition-based training drills. Future research should aim to understand the relationship between HAE load and concussion diagnosis, and how HAEs occur in training. Additionally, understanding what specific training drills players take part in during a typical week could support an understanding of how to effectively manage individual players, prescribing the minimum HAE dosage for competition preparation on an individual basis. Practitioners might wish to use this data as a benchmark when interpreting and managing the individual players’ HAE exposure.

## Supplementary Information

Below is the link to the electronic supplementary material.

Supplementary file1 (PDF 737 KB)

## Declarations

### Availability of data and material

All data relevant to the study are included in the article or uploaded as supplementary information. Anonymised data should be available upon reasonable request.
