# Supplementary material for: Head Acceleration Event Exposure During Elite Men’s and Women’s Rugby Union Training
Source: Sports Med. 2025 Aug 2;56(1):229–41. doi: 10.1007/s40279-025-02287-2 (PMC12913257; doi:10.1007/s40279-025-02287-2)
Supplement: Supplementary file 1 — Supplementary file1 (PDF 737 KB) [file 40279_2025_2287_MOESM1_ESM.pdf]

Title: Head Acceleration Event Exposure during Elite Men's and Women's Rugby Union Training

Journal: Sports Medicine

Authors: Samuel Hudson[1,2], James Tooby[3], Gregory Roe[3], Thomas Sawczuk[3,4], Dario Cazzola[1,2], Matt Cross[3,5], Ben Jones[3,5,6,7,8]. Simon Kemp[9,10], Sarah Whitehead[3], Keith Stokes[1,2,9]

Affiliations:

[1] Centre for Health and Injury and Illness Prevention in Sport, University of Bath, United Kingdom

[2] UK Collaborating Centre on Injury and Illness Prevention in Sport (UKCCIIS), University of Bath, United Kingdom

[3]Carnegie Applied Rugby Research (CARR) centre, Carnegie School of Sport, Leeds Beckett University, Leeds, UK.

[4]Obesity Institute, Leeds Beckett University, Leeds, UK.

[5]Premiership Rugby, London, UK.

[6]Division of Physiological Sciences, Department of Human Biology, Faculty of Health Sciences, University of Cape Town, Cape Town, South Africa

[7]School of Behavioural and Health Sciences, Faculty of Health Sciences, Australian Catholic University, Brisbane, QLD, Australia

[8]Rugby Football League, Etihad Campus, Manchester, United Kingdom

[9]Rugby Football Union, Twickenham, UK.

[10]Department of Epidemiology and Population Health, London School of Hygiene and Tropical Medicine, London, UK

Corresponding author: Samuel Hudson, sjh241@bath.ac.uk

Level of Contact	HAE Count					Players Sampled (*n*)	Player Exposures (*n*)	Minutes of Player Exposure	HAE Incidence (*per player minute & 95% CI*)				
	≥5 *g*	≥10 *g*	≥15 *g*	≥20 *g*	≥25 *g*				≥5 *g*	≥10 *g*	≥15 *g*	≥20 *g*	≥25 *g*
**Men's Forwards**													
**Full Contact**	**1469**	**977**	**474**	**227**	**104**	**138**	**1895**	**8392**	**0.20 (0.16 - 0.26)**	**0.12 (0.10 - 0.16)**	**0.06 (0.05 - 0.08)**	**0.03 (0.02 - 0.04)**	**0.02 (0.01 - 0.03)**
Live Backs Units	-	-	-	-	-	-	-	-	-	-	-	-	-
Live Lineout	881	602	289	129	57	135	855	3027	0.32 (0.26 - 0.40)	0.21 (0.17 - 0.29)	0.11 (0.08 - 0.15)	0.05 (0.04 - 0.09)	0.03 (0.02 - 0.05)
Live Scrum	67	34	14	6	5	124	744	2544	0.02 (0.02 - 0.04)	0.01 (0.01 - 0.02)	0.01 (0.00 - 0.02)	0.00 (0.00 - 0.01)	0.00 (0.00 0.01)
Team Full Contact	521	341	171	92	42	70	2820	296	0.20 (0.17 - 0.26)	0.13 (0.10 - 0.17)	0.07 (0.05 - 0.09)	0.04 (0.03 - 0.06)	0.02 (0.01 - 0.04)
**Controlled Contact**	**2677**	**1600**	**724**	**341**	**161**	**146**	**3197**	**25724**	**0.09 (0.08 - 0.10)**	**0.05 (0.05 - 0.06)**	**0.02 (0.02 - 0.03)**	**0.01 (0.01 - 0.01)**	**0.01 (0.00 - 0.01)**
Live Breakdown	92	58	30	15	8	28	122	68	0.79 (0.60 - 1.02)	0.54 (0.38 - 0.76)	0.34 (0.20 - 0.53)	0.17 (0.08 - 0.29)	0.08 (0.03 - 0.20)
Live Tackle Drill	158	81	39	14	10	66	213	629	0.28 (0.22 - 0.37)	0.14 (0.10 - 0.19)	0.07 (0.05 - 0.12)	0.03 (0.01 - 0.05)	0.02 (0.01 - 0.03)
Team Shoulder On	2016	1203	547	255	119	138	1849	21817	0.09 (0.07 - 0.10)	0.05 (0.04 - 0.06)	0.02 (0.02 - 0.03)	0.01 (0.01 - 0.01)	0.01 (0.00 - 0.01)
Wrestling	20	14	7	4	3	5	11	23	0.63 (0.16 - 1.39)	0.44 (0.03 - 0.91)	0.22 (0.00 - 0.44)	0.13 (0.00 - 0.31)	0.09 (0.00 - 0.19)
Breakdown Bags (Opposed)	95	51	21	10	1	91	252	759	0.16 (0.12 - 0.21)	0.08 (0.05 - 0.11)	0.04 (0.02 - 0.07)	0.02 (0.01 - 0.03)	0.00 (0.00 - 0.02)
Breakdown Bags (Unopposed)	21	10	3	1	0	44	91	258	0.09 (0.05 - 0.16)	0.05 (0.02 - 0.12)	0.02 (0.00 - 0.09)	0.02 (0.00 - 0.07)	0.00 (NA)
Lineout vs Pads	52	40	20	10	6	28	100	299	0.21 (013 - 0.36)	0.17 (0.10 - 0.30)	0.07 (0.04 - 0.12)	0.03 (0.01 - 0.05)	0.02 (0.01 - 0.04)
Sled Scrum	2	1	0	0	0	44	73	324	0.00 (0.00 - 0.01)	0.00 (0.00 - 0.01)	0.00 (NA)	0.00 (NA)	0.00 (NA)
Tackle Bags Drill	161	104	42	23	12	94	212	462	0.36 (0.28 - 0.46)	0.23 (0.17 - 0.32)	0.10 (0.06 - 0.18)	0.06 (0.03 - 0.12)	0.02 (0.01 - 0.04)
Team vs Pads	60	38	15	9	2	83	1174	317	0.05 (0.03 - 0.07)	0.03 (0.02 - 0.05)	0.01 (0.01 - 0.02)	0.01 (0.00 - 0.02)	0.00 (0.00 - 0.01)

**Non Contact**	**143**	**76**	**43**	**15**	**4**	**131**	**994**	**5100**	**0.03 (0.02 - 0.05)**	**0.01 (0.01 - 0.02)**	**0.01 (0.01 - 0.02)**	**0.00 (0.00 - 0.01)**	**0.00 (0.00 - 0.00)**
Unopposed Backs Units	-	-	-	-	-	-	-	-	-	-	-	-	-
Unopposed Lineout	108	61	39	14	4	124	621	3593	0.03 (0.02 - 0.04)	0.14 (0.01 - 0.22)	0.01 (0.01 - 0.02)	0.00 (0.00 - 0.01)	0.00 (0.00 - 0.00)
Passing	8	3	1	1	0	32	138	432	0.01 (0.01 - 0.03)	0.00 (0.00 - 0.01)	0.00 (0.00 - 0.00)	0.00 (0.00 - 0.00)	0.00 (NA)
Team Unopposed Organisation	27	12	3	0	0	78	246	1112	0.03 (0.01 - 0.06)	0.01 (0.00 - 0.02)	0.00 (0.00 - 0.02)	0.00 (NA)	0.00 (NA)
**Men's Backs**													
**Full Contact**	**183**	**125**	**53**	**25**	**14**	**40**	**156**	**1648**	**0.16 (0.09 - 0.41)**	**0.10 (0.06 - 0.26)**	**0.03 (0.02 - 0.04)**	**0.01 (0.01 - 0.02)**	**0.01 (0.00 - 0.01)**
Live Backs Units	23	20	7	2	2	10	13	30	0.89 (0.40 - 1.60)	0.73 (0.34 - 1.23)	0.21 (0.07 - 0.49)	0.06 (0.03 - 0.11)	0.06 (0.00 - 0.22)
Live Lineout	-	-	-	-	-	-	-	-	-	-	-	-	-
Live Scrum	-	-	-	-	-	-	-	-	-	-	-	-	-
Team Full Contact	160	1105	46	23	12	38	143	1617	0.09 (0.07 - 0.13)	0.06 (0.04 - 0.07)	0.02 (0.01 - 0.04)	0.01 (0.01 - 0.02)	0.01 (0.00 - 0.01)
**Controlled Contact**	**836**	**482**	**225**	**122**	**61**	**90**	**1320**	**10975**	**0.11 (0.08 - 0.20)**	**0.07 (0.05 - 0.12)**	**0.03 (0.02 - 0.05)**	**0.02 (0.01 - 0.03)**	**0.01 (0.00 - 0.02)**
Live Breakdown	67	47	17	9	5	15	37	114	0.51 (0.32 - 0.77)	0.37 (0.25 - 0.53)	0.11 (0.06 - 0.18)	0.06 (0.03 - 0.11)	0.04 (0.02 - 0.08)
Live Tackle Drill	81	54	24	14	8	35	83	253	0.27 (0.18 - 0.38)	0.19 (0.12 - 0.32)	0.09 (0.05 - 0.18)	0.06 (0.03 - 0.16)	0.03 (0.00 - 0.11)
Team Shoulder On	562	312	152	85	43	87	799	9519	0.09 (0.06 - 0.20)	0.05 (0.03 - 0.11)	0.03 (0.02 - 0.05)	0.02 (0.01 - 0.03)	0.01 (0.00 - 0.03)
Wrestling	-	-	-	-	-	-	-	-	-	-	-	-	-
Breakdown Bags (Opposed)	47	30	14	5	3	50	129	292	0.14 (0.08 - 0.27)	0.10 (0.05 - 0.25)	0.07 (0.02 - 0.20)	0.04 (0.01 - 0.10)	0.01 (0.00 - 0.04)
Breakdown Bags (Unopposed)	13	7	3	1	0	21	34	91	0.13 (0.05 - 0.26)	0.05 (0.01 - 0.14)	0.02 (0.00 - 0.07)	0.00 (0.00 - 0.02)	0.00 (NA)
Lineout vs Pads	-	-	-	-	-	-	-	-	-	-	-	-	-
Sled Scrum	-	-	-	-	-	-	-	-	-	-	-	-	-
Tackle Bags Drill	55	29	12	6	2	53	110	232	0.26 (0.18 - 0.40)	0.15 (0.10 - 0.22)	0.08 (0.03 - 0.15)	0.03 (0.01 - 0.11)	0.02 (0.00 - 0.08)

Team vs Pads	11	3	3	2	0	37	108	414	0.03 (0.01 - 0.06)	0.01 (0.00 - 0.01)	0.01 (0.00 - 0.02)	0.00 (0.00 - 0.01)	0.00 (NA)
**Non Contact**	**112**	**46**	**20**	**10**	**6**	**66**	**598**	**4613**	**0.02 (0.01 - 0.03)**	**0.01 (0.00 - 0.01)**	**0.00 (0.00 - 0.01)**	**0.00 (0.00 - 0.01)**	**0.00 (0.00 - 0.00)**
Unopposed Backs Units	86	35	16	8	5	63	391	3743	0.02 (0.01 - 0.03)	0.01 (0.00 - 0.01)	0.00 (0.00 - 0.01)	0.00 (0.00 - 0.01)	0.00 (0.00 - 0.00)
Unopposed Lineout	-	-	-	-	-	-	-	-	-	-	-	-	-
Passing	9	3	0	0	0	21	98	339	0.02 (0.00 - 0.04)	0.00 (0.00 - 0.01)	0.00 (NA)	0.00 (NA)	0.00 (NA)
Team Unopposed Organisation	17	8	4	2	1	39	129	590	0.02 (0.01 - 0.04)	0.01 (0.00 - 0.03)	0.00 (0.00 - 0.01)	0.00 (0.00 - 0.01)	0.00 (0.00 - 0.00)

Level of Contact	HAE Count					Players Sampled (*n*)	Player Exposures (*n*)	Minutes of Player Exposure	HAE Incidence (*per player minute & 95% CI*)				
	≥5 *g*	≥10 *g*	≥15 *g*	≥20 *g*	≥25 *g*				≥5 *g*	≥10 *g*	≥15 *g*	≥20 *g*	≥25 *g*
**Women's Forwards**													
**Full Contact**	**193**	**111**	**48**	**19**	**12**	**67**	**219**	**1733**	**0.10 (0.08 - 0.14)**	**0.06 (0.04 - 0.08)**	**0.03 (0.02 - 0.04)**	**0.01 (0.00 - 0.02)**	**0.01 (0.00 - 0.01)**
Live Backs Units	-	-	-	-	-	-	-	-	-	-	-	-	-
Live Lineout	51	28	14	4	3	48	476	348	0.12 (0.09 - 0.18)	0.05 (0.03 - 0.08)	0.03 (0.02 - 0.05)	0.01 (0.00 - 0.02)	0.01 (0.00 - 0.02)
Live Scrum	4	0	0	0	0	42	522	95	0.00 (0.00 - 0.01)	0.00 (NA)	0.00 (NA)	0.00 (NA)	0.00 (NA)
Team Full Contact	138	83	34	15	9	32	46	735	0.22 (0.14 - 0.34)	0.15 (0.09 - 0.28)	0.06 (0.03 - 0.12)	0.03 (0.01 - 0.07)	0.02 (0.00 - 0.06)
**Controlled Contact**	**463**	**250**	**89**	**36**	**14**	**88**	**1273**	**9969**	**0.05 (0.04 - 0.06)**	**0.03 (0.02 - 0.03)**	**0.01 (0.01 - 0.01)**	**0.00 (0.00 - 0.01)**	**0.00 (0.00 - 0.00)**
Live Breakdown	-	-	-	-	-	-	-	-	-	-	-	-	-
Live Tackle Drill	84	54	16	8	0	64	159	799	0.14 (0.11 - 0.20)	0.09 (0.07 - 0.14)	0.02 (0.01 - 0.04)	0.01 (0.01 - 0.03)	0.00 (NA)
Team Shoulder On	298	152	61	24	12	72	802	7718	0.04 (0.03 - 0.05)	0.02 (0.02 - 0.03)	0.01 (0.01 - 0.02)	0.00 (0.00 - 0.01)	0.00 (0.00 - 0.00)
Wrestling	2	0	0	0	0	2	2	7	0.29 (NA)	0.00 (NA)	0.00 (NA)	0.00 (NA)	0.00 (NA)

Breakdown Bags (Opposed)	29	16	5	2	1	39	78	348	0.07 (0.04 - 0.11)	0.04 (0.03 - 0.07)	0.02 (0.00 - 0.03)	0.00 (0.00 - 0.01)	0.00 (0.00 - 0.02)
Breakdown Bags (Unopposed)	2	0	0	0	0	30	41	176	0.01 (0.00 - 0.03)	0.00 (NA)	0.00 (NA)	0.00 (NA)	0.00 (NA)
Lineout vs Pads	7	3	1	0	0	25	33	149	0.06 (0.02 - 0.14)	0.02 (0.00 - 0.08)	0.00 (0.00 - 0.02)	0.00 (NA)	0.00 (NA)
Sled Scrum	-	-	-	-	-	-	-	-	-	-	-	-	-
Tackle Bags Drill	25	17	1	1	1	36	96	297	0.09 (0.06 - 0.15)	0.07 (0.04 - 0.11)	0.00 (0.00 - 0.01)	0.00 (0.00 - 0.01)	0.00 (0.00 - 0.01)
Team vs Pads	16	8	5	1	0	42	62	476	0.04 (0.01 - 0.08)	0.03 (0.01 - 0.08)	0.02 (0.00 - 0.06)	0.00 (0.00 - 0.00)	0.00 (NA)
**Non Contact**	**45**	**27**	**12**	**7**	**4**	**157**	**377**	**4187**	**0.01 (0.01 - 0.01)**	**0.01 (0.00 - 0.01)**	**0.00 (0.00 - 0.00)**	**0.00 (0.00 - 0.00)**	**0.00 (0.00 - 0.00)**
Unopposed Backs Units	-	-	-	-	-	-	-	-	-	-	-	-	-
Unopposed Lineout	17	10	5	3	1	58	135	1755	0.01 (0.00 - 0.02)	0.01 (0.00 - 0.02)	0.00 (0.00 - 0.01)	0.00 (0.00 - 0.00)	0.00 (0.00 - 0.00)
Passing	4	3	0	0	0	33	49	257	0.02 (0.00 - 0.04)	0.01 (0.00 - 0.04)	0.00 (NA)	0.00 (NA)	0.00 (NA)
Team Unopposed Organisation	24	13	7	4	3	66	193	2175	0.01 (0.01 - 0.03)	0.01 (0.00 - 0.02)	0.00 (0.00 - 0.01)	0.00 (0.00 - 0.00)	0.00 (0.00 - 0.00)
**Women's Backs**													
**Full Contact**	**29**	**15**	**10**	**3**	**1**	**16**	**33**	**586**	**0.05 (0.03 - 0.11)**	**0.03 (0.01 - 0.09)**	**0.03 (0.01 - 0.09)**	**0.02 (0.00 - 0.08)**	**0.02 (0.00 - 0.05)**
Live Backs Units	5	1	1	0	0	7	7	61	0.08 (0.02 - 0.18)	0.02 (0.00 - 0.03)	0.02 (0.00 - 0.05)	0.00 (NA)	0.00 (NA)
Live Lineout	-	-	-	-	-	-	-	-	-	-	-	-	-
Live Scrum	-	-	-	-	-	-	-	-	-	-	-	-	-
Team Full Contact	24	14	9	3	1	16	26	524	0.05 (0.03 - 0.10)	0.03 (0.01 - 0.09)	0.03 (0.01 - 0.08)	0.02 (0.00 - 0.08)	0.02 (0.00 - 0.05)
**Controlled Contact**	**115**	**59**	**26**	**6**	**3**	**54**	**452**	**3416**	**0.05 (0.03 - 0.07)**	**0.03 (0.02 - 0.04)**	**0.01 (0.01 - 0.02)**	**0.00 (0.00 - 0.01)**	**0.00 (0.00 - 0.00)**
Live Breakdown	-	-	-	-	-	-	-	-	-	-	-	-	-
Live Tackle Drill	46	21	10	0	0	37	83	383	0.10 (0.06 - 0.14)	0.05 (0.03 - 0.07)	0.02 (0.01 - 0.04)	0.00 (NA)	0.00 (NA)
Team Shoulder On	52	26	13	6	3	42	275	2594	0.03 (0.02 - 0.05)	0.02 (0.01 - 0.04)	0.01 (0.00 - 0.02)	0.00 (0.00 - 0.01)	0.00 (0.00 - 0.01)

Wrestling	-	-	-	-	-	-	-	-	-	-	-	-	-
Breakdown Bags (Opposed)	11	10	3	0	0	24	50	211	0.06 (0.03 - 0.10)	0.06 (0.03 - 0.10)	0.02 (0.00 - 0.06)	0.00 (NA)	0.00 (NA)
Breakdown Bags (Unopposed)	0	0	0	0	0	2	2	10	0.00 (NA)	0.00 (NA)	0.00 (NA)	0.00 (NA)	0.00 (NA)
Lineout vs Pads	-	-	-	-	-	-	-	-	-	-	-	-	-
Sled Scrum	-	-	-	-	-	-	-	-	-	-	-	-	-
Tackle Bags Drill	6	2	0	0	0	18	29	89	0.11 (0.02 - 0.40)	0.08 (0.00 - 0.41)	0.00 (NA)	0.00 (NA)	0.00 (NA)
Team vs Pads	0	0	0	0	0	8	13	130	0.00 (NA)	0.00 (NA)	0.00 (NA)	0.00 (NA)	0.00 (NA)
**Non Contact**	**39**	**27**	**18**	**12**	**5**	**38**	**178**	**1988**	**0.04 (0.02 - 0.08)**	**0.02 (0.01 - 0.05)**	**0.01 (0.00 - 0.04)**	**0.01 (0.00 - 0.03)**	**0.00 (0.00 - 0.01)**
Unopposed Backs Units	29	24	16	12	5	33	80	820	0.05 (0.02 - 0.09)	0.03 (0.01 - 0.07)	0.01 (0.00 - 0.05)	0.01 (0.00 - 0.04)	0.01 (0.00 - 0.02)
Unopposed Lineout	-	-	-	-	-	-	-	-	-	-	-	-	-
Passing	2	0	0	0	0	7	15	79	0.03 (0.00 - 0.14)	0.00 (NA)	0.00 (NA)	0.00 (NA)	0.00 (NA)
Team Unopposed Organisation	8	3	2	0	0	30	83	1089	0.01 (0.01 - 0.03)	0.00 (0.00 - 0.00)	0.00 (0.00 - 0.00)	0.00 (NA)	0.00 (NA)

Table S1: Exposure data and average HAE incidence per player minute (CI: 95%) for men’s and women’s forwards and backs during each level of contact training and drill type at different PLA thresholds.

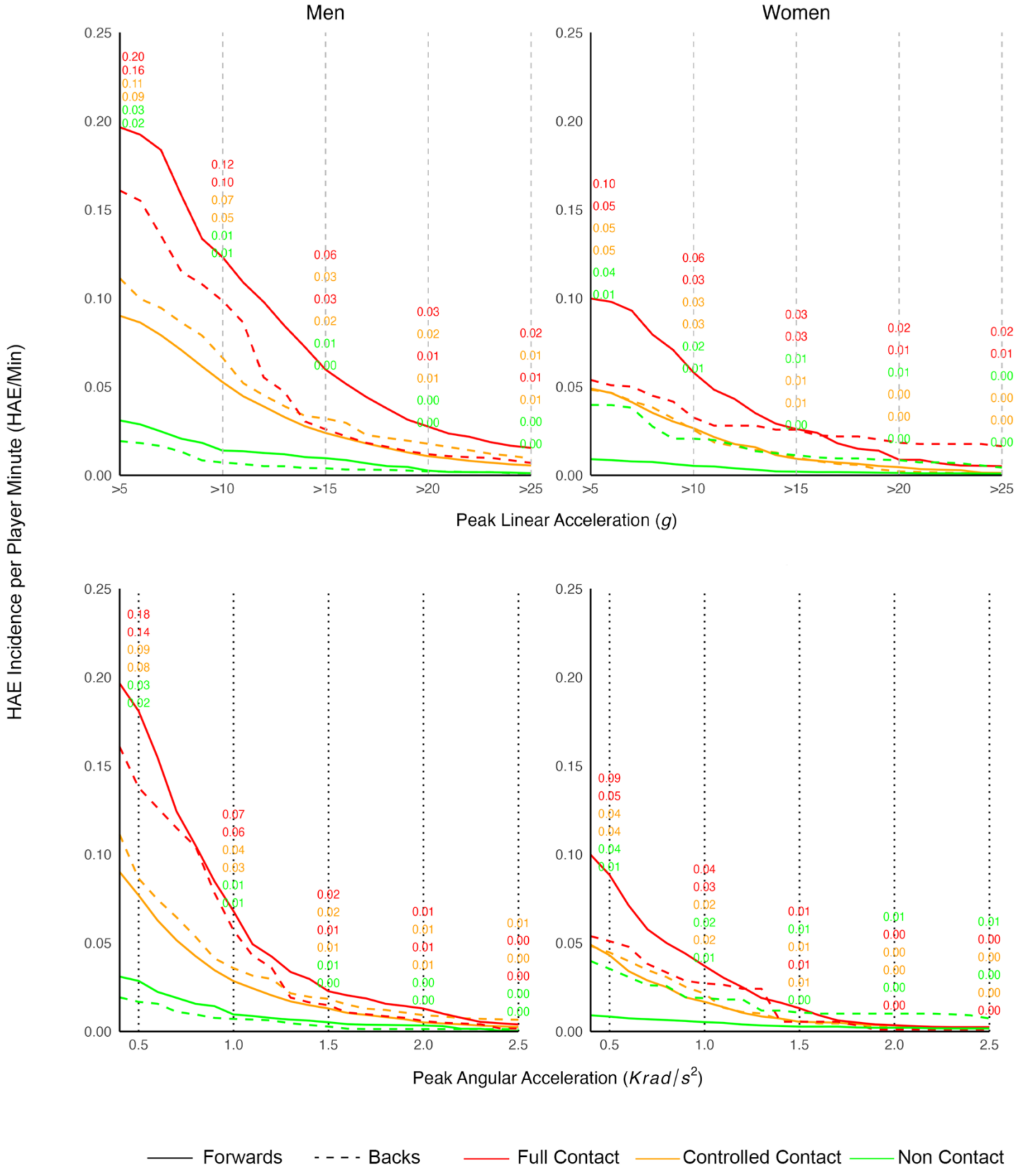

Figure S1: HAE incidence per player minute for men's and women's forwards and backs in contact training at different PLA and PAA magnitude thresholds.

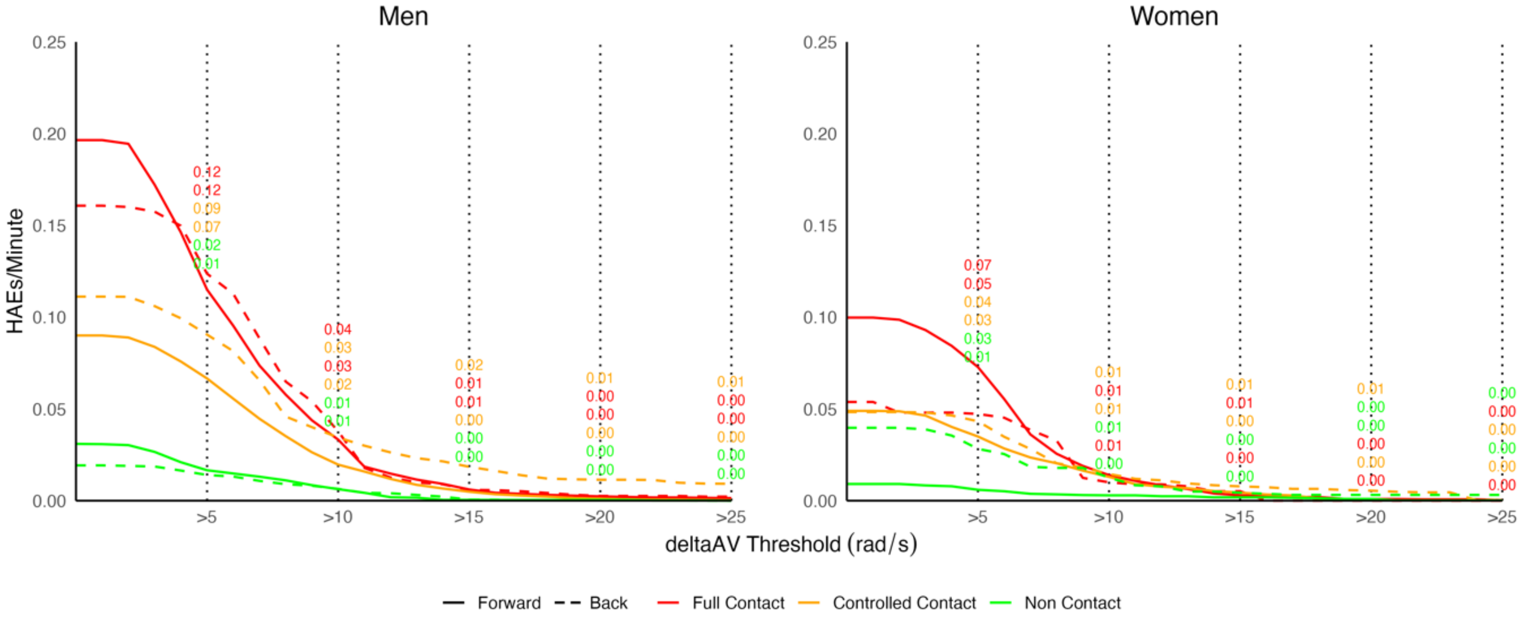

Figure S2: HAE incidence per player minute for men's and women's forwards and backs in contact training at different delta angular velocity magnitude thresholds.
